# Modeling Rare Species Distribution at the Edge: The Case for the Vulnerable Endemic Pyrenean Desman in France

**DOI:** 10.1100/2012/612965

**Published:** 2012-04-19

**Authors:** M. Williams-Tripp, F. J. N. D'Amico, C. Pagé, A. Bertrand, M. Némoz, J. A. Brown

**Affiliations:** ^1^Department of Mathematics and Statistics, University of Canterbury, Private Bag 4800, Christchurch, New Zealand; ^2^Université de Pau & Pays de l'Adour, UFR Sciences & Techniques Côte Basque, Campus Montaury, 64600 Anglet, France; ^3^INRA UMR ECOBIOP, Quartier Ibarron, 64310 Saint-Pée sur Nivelle, France; ^4^SUC au CERFACS, URA CERFACS/CNRS no.1875, 42 Avenue Gaspard Coriolis, 31057 Toulouse Cedex 01, France; ^5^ABELA, 09320 Boussenac, France; ^6^Conservatoire Régional des Espaces Naturels de Midi-Pyrénées, 75, voie du Toec, BP 57611, 31076 Toulouse Cedex 3, France

## Abstract

The endemic Pyrenean Desman (*Galemys pyrenaicus*) is an elusive, rare, and vulnerable species declining over its entire and narrow range (Spain, Portugal, France, and Andorra). The principal set of conservation measures in France is a 5-years National Action Plan based on 25 conservation actions. Priority is given to update its present distribution and develop tools for predictive distribution models. We aim at building the first species distribution model and map for the northern edge of the range of the Desman and confronting the outputs of the model to target conservation efforts in the context of environmental change. Contrasting to former comparable studies, we derive a simpler model emphasizing the importance of factors linked to precipitation and not to the temperature. If temperature is one of the climate change key factors, depicted shrinkage in Desman distribution could be lower or null at the northern (French) edge suggesting thus a major role for this northern population in terms of conservation of the species. Finally, we question the applied issue of temporal and spatial transferability for such environmental favourability models when it is made at the edge of the distribution range.

## 1. Introduction

Despite some lack of information, present knowledge suggests that populations of the endemic Pyrenean Desman (*Galemys pyrenaicus*) are decreasing over the entire distribution area that is, Spain, Portugal, France and Andorra (see distribution map on the IUCN Redlist website at http://www.iucnredlist.org/apps/redlist/details/8826/0/rangemap), even undergoing a stronger decline in the former country according to Fernandes et al. [3]. Pyrenean Desman are thus listed as Vulnerable (Vu A2ac + 3c + 4ac) under the IUCN criteria [4] and in the national Portuguese and Spanish Red Lists [5]. The species may be locally considered in Critical Danger [6] and was mentioned as “Near Threatened” in the French Red List [7]. Worries about the present status of this species and fears for the future (further expected realistic decline of at least 30% over the next ten years [3]) have led to adoption of diverse conservation actions in Portugal [8], Spain [9] and France [10]. The French National Action Plan (NAP) for Desman runs from 2010 to 2015 and proposes 25 conservation actions organized in different objectives under three main headings: studies, conservation, and communication. One of the priority actions (Action 2 under Objective I-A) is to define and standardize survey tools for the study of the present and future distribution of the species in its French range [10].

Effective and efficient development of such NAP priority actions that involve monitoring and conservation management require both field surveys and predictive or species distribution modelling. Field surveys with either direct assessment of individuals, or species-presence surveys provide information on the actual distribution [11, 12], but are limited by inherent difficulties such as nondetection [13] and by the even more prohibitively problem nowadays of the cost in time and/or money [14]. Thus, for Desman, as for almost all rare and elusive species, production of good distribution models to supplement field surveys is crucial and mandatory. Distribution models are not without their limits often linked to the rarity and elusiveness of the species itself, a paradoxical situation called the “rare species modelling paradox” by Lomba et al. [15].

Species distribution models (SDMs) are popular tools in ecology because of their usefulness in conservation biology (for a review see [16, 17]). A good model will capture the correlation between environmental factors and the distribution of the studied species and can be used to assess the importance of environmental specific factors and/or to predict species' distribution across unsampled areas in the natural species range and to examine environmental change consequences [17]. One of the important interests of SDMS is their transferability that is, their applicability across different spatiotemporal patterns [18, 19].

By contrast to the Desman conservation efforts in the Iberian Peninsula area, where comprehensive specific field presence-absence surveys [6, 8] do coexist with model-based approaches [1, 20], no predictive distribution model or SDMs is available yet for France. This limits, for example, any possibility of detecting range shifts [21] or possible changes in population declines or recovery under usual environmental scenario. SDM for Desman have been made available very recently for the whole Iberian Peninsula as well as for its two countries (Portugal and Spain) independently by two teams and at the same scale. Barbosa et al. [1] used a favourability function with a GLM approach [22] to provide maps with 10 km × 10 km favourability values (range 0–1), whilst Morueta-Holme et al. [20] chose a machine-learning method using MAXENT (maximum entropy) to produce maps with estimates of the probability of presence (range 0–1) conditioned on the environmental variables in each 10 km × 10 km grid cell.

In this study, we used SDM transferability as a guide for projecting Barbosa et al. [1] model into the remaining unknown northern area (transferability in space) of the species and lay the basis for future predictions of climate-change responses (transferability in time) as Morueta-Holme et al. [20] intend. The aim of our work is thus to produce for the first time an SDM for the northern edge (French part) of the range of this species and to confront the outputs of the model to the species presence-absence data gathered over the recent period to produce an updated image of the actual distribution of the Desman in France, at the northern edge of its actual natural range. This is a crucial step in order to fill the last gap in Desman distribution modelling and enable for conservation efforts at the scale of the whole distribution area of this endemic vulnerable species. We also discuss the applied issue of temporal and spatial transferability for such environmental favourability models when it is made at the edge of the distribution range of a rare species.

## 2. Methods

### 2.1. Data Collection

We used a comprehensive set of species-presence data (i.e., records of where the species was observed) gathered from a number of different surveys. Large scale surveys for Pyrenean Desman were conducted between 1985 and 1990, using the same field methodology (searching for scats within the channel on emerging rocks and on river banks, along 500 m river stretches and stopping if scats found) and observer throughout the French part of the Pyrenees. Medium- and fine-scale surveys were conducted in Ariège and Haute-Garonne counties, also with the same field methodology. This dataset consists of 1576-point data (A. Bertrand, unpublished), and was used to map the known distribution of the Desman in its French range. Despite some scale heterogeneity in field data collection, the prevalence of nondetection should not vary among surveys because they were all conducted by the same observer [23].

The meteorological data used in our work came from the output of a statistical downscaling methodology (dsclim) that has been developed to study climate and downscale output from large-scale reanalysis and global climate models [24, 25] and applied, notably and successfully, to hydrology, agronomy, and France mountainous areas climate studies. The methodology is based on links between the large-scale atmospheric circulation and the local climate. It is able to reproduce the main characteristics of the climate (inter-annual variability, average, etc.). The methodology performs a resampling of observed days from a “training period” and classified by weather types (e.g., days having similar atmospheric circulations). The training was performed using the National Center for Environmental Prediction (NCEP) reanalysis [26] and the Météo-France SAFRAN mesoscale meteorological analysis [27]. The dsclim methodology was applied using the NCEP reanalysis large-scale atmospheric mean sea-level pressure over the period 1990–2000 to generate averaged values for the whole period for the following meteorological parameters at an 8-km spatial resolution: mean annual precipitation, mean annual temperature, mean annual number of days with precipitation greater than or equal to 0.1 mm, mean relative atmosphere humidity in July at 07 h, and mean temperature in January.

### 2.2. Modelling Methodology

We used a favourability function with a GLM approach (full description in [1, 22]) to model Desman distribution in France and produce environmental favourability maps with 8 km × 8 km favourability values. Following Barbosa et al. [1], we extrapolated the Spanish model to French terrain and built 2 new French distribution models using the 8 environmental and spatial predictor variables (Table 1) identified in the Spanish model as optimal for transference performance. Slight modifications were made for solar radiation data (see erratum in [2]). Of the new models, one GLM approach included latitude, as well as the environmental predictor variables for input, while a second GLM approach allowed only environmental predictor variables. Assessment of model's accuracy and performance was made using a set of selected indices (definitions in [17, 28, 29]) with emphasis on discrimination capacity and not reliability [30]. Among the available threshold-dependent indices specificity, we chose the following simple and intuitive measures for the model's performance: sensitivity, specificity, percent correctly classified (PCC also called overall accuracy (OA)), proportion of predicted present correctly predicted (PPP), proportion of predicted absent correctly predicted (NPP), and true skill statistic (TSS). Compared with the widely used Kappa index, the latter presents the advantage of being independent of prevalence [28]. The well-used area under the ROC curve (AUC) was used as a threshold-independent index with the further advantage of also being independent of prevalence.

## 3. Results and Discussion

**Figure 1:**
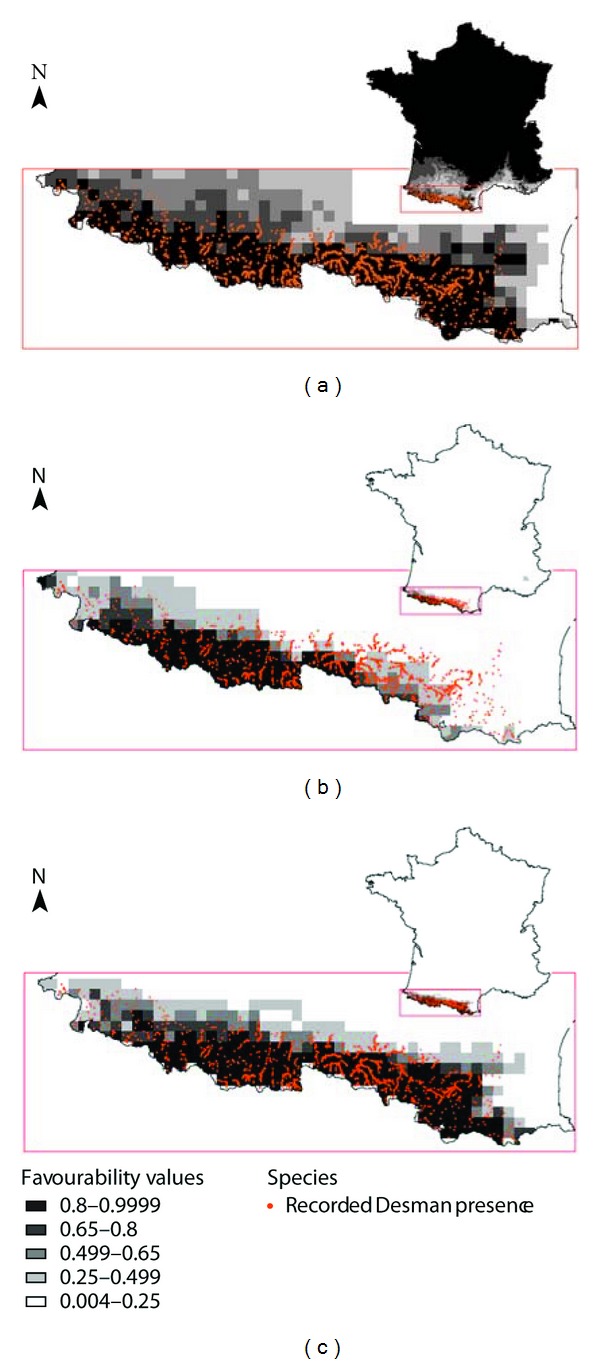
Superposed known distribution (Presence only—orange dots) of *Galemys pyrenaicus* and environmental favourability map (favourability values ranging from 0 to 1) for the French part of the species range as given by the 3 models chosen (selected variables and coefficients in Table 1): (a) Transferred environmental favourability model described by Barbosa et al. [1] under the name “Spanish model” and corrected for SRad range error (see erratum by Barbosa et al. [2]). (b) newly proposed model (French model without “Latitude”). (c) newly proposed model (French model with “Latitude”).

**Figure 2:**
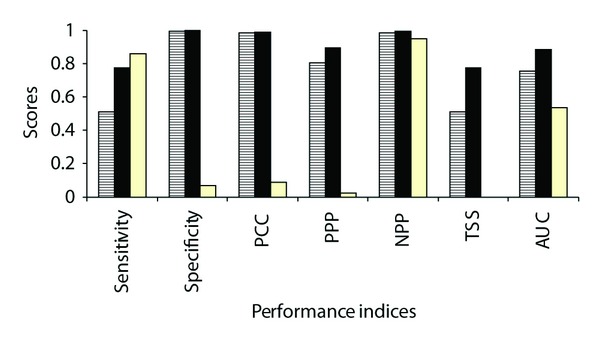
Performance scores for each environmental favourability model: sensitivity, specificity, percent correctly classified (PCC), proportion of predicted present correctly predicted (PPP), proportion of predicted absent correctly predicted (NPP), true skill statistic (TSS), and Area under the ROC function. (Legend: black and white stripes: French model without “Latitude”; black bars: (French model with “Latitude”; white bars: “Spanish model”).

This paper brings for the first time environmental and geographical information on the distribution of Desman in its northern marginal distribution, as expressed by SDM. The three tested models for the French part of Desman range produced very different predictions (Figure 1) with marked difference in performance (Figure 2). The Spanish model, described as the better predictive one among the so far tested predictive models by Barbosa et al. [1] and thus the best candidate for transference to other geographical areas, displayed very poor predictive ability for the French range of the species. For the Spanish model, PCC and PPP were far lower than for French models whereas NPP was comparable and sensitivity higher; true skill statistics (TSS) was negative in the case of the Spanish model, indicating a performance no better than random [28]. The AUC value was lower in the Spanish model (0.534) compared with the two French models (0.754 and 0.887). The French models had higher discrimination capacity, with the simplest one (5 variables including latitude) achieving a high level of performance. The latitude factor proved to be a key factor despite the fact that its importance was not immediately apparent at the step of conceiving the study. Excluding latitude (keeping “mean summer relative humidity” (*HJul*) and “mean annual temperature” (*Temp*) as in the Spanish model) skewed the distribution and underestimated favourability in the eastern part of the French Pyrenean area. The best predictive model for the French part, at the northern edge of the natural Desman range, was thus a simpler model using only 5 variables (compared to the 8 variables required in the Spanish one Table 1).

The preferred model emphasized the importance of factors linked to precipitation (“Mean annual precipitation” *Prec* and “Mean annual number of days with precipitation >0.1 mm” *DPre*) and not to the temperature. This contrasts with the key results of Barbosa et al. [1] and even those of the Maxent-derived approach used by Morueta-Holme et al. [20], where both teams concluded that summer temperature, combined with precipitation characteristics, had a critical role in influencing the distribution of the species in the Iberian Peninsula.

The second main conclusion of this study is the lack of transferability for the Spanish environmental favourability model, contrasting with Barbosa et al. [1] assertion that this model could have high transferability and thus potential conservation issues. Indeed, failure to achieve full transferability in space or marked asymmetry in transferability between different regions is not puzzling and has been documented for many species. Numerous recent contributions and reviews have listed and analysed reasons why SDMs fail sometimes in being effectively transferable [17, 19, 31]. Apart from the considerable variation in the transferability of SDMs between modelling techniques addressed in these papers [31, 32], the depicted pitfalls for regional variation fall in two main groups [19]: (1) peculiarities of these regions within the species natural range, involving for example differences in the ranges of environmental predictors [29] or the varied impact of land-use history [33] and (2) species-specific reasons like differential phenotypic plasticity or existence of ecotypes [34].

We hypothesize that large-scale effects, mainly the geographical situation of the study areas at the edge of the present distribution range of the species, affected transferability. Indeed, the prominent contrast in the physical/climatic characteristics of the tiny northern part of the range (rainy north-facing slopes of the French Pyrénées dominated by Atlantic climatic influence), and the large southern part of the species range (drier south-facing slopes of the South Pyrénées, under Mediterranean climatic influence) is prone to impinge on the relative influence of selected factors (mainly precipitation and temperature) for the models and thus influence transferability. We cannot exclude the possibility that other large-scale effects (such as biological quality of rivers, importance of fully protected areas in each country, watershed fragmentation by hydro-electrical power schemes, and differential flow management) or fine scales effects (such as physical features of the river habitat at channel/bank level or biotic interactions) are at work. Yet, the so-far described models could still fail at capturing the actual environmental factors explaining present distribution of the Desman. Eventually, one cannot exclude that differential phenotypic plasticity or ecotypes exist. We could speculate that a “northern” ecotype exists at the northern marginal edge in the French Pyrénées and that little genetic exchange with another “southern” ecotype in the Iberian Peninsula occurs, something that can be tested properly with the genetic tools available [35]. Both hypothesis (north-south contrast and existence of ecotypes) are supported by the fact that our findings emphasize the importance of factors linked to the regime of precipitation and not to the temperature. In the context of the global climate change, assuming that temperature is one of the key factors at work, expected shrinkage in Desman distribution [20] could be lower or null at the French edge suggesting thus a different trajectory for the French population of Desman and a key role in terms of conservation of the species. If true, it could by consequence partly challenge recent claims on Desman range shift following climate change and totally invalidate proposed assisted migration for this species [20, 36].

To conclude, bearing in mind that the SDM literature is not yet completely established at least to the necessary operational state providing clear guidance for selecting relevant methods [31], our GLM study along with that of Barbosa et al. [1] add now to supply a comprehensive SDM-derived map of the present whole-range Desman distribution. It provides better opportunity to revisit range-shift scenarios especially at northern limits of the species range, explore the possible existence of differential phenotypic plasticity or ecotypes, and monitor this rare species at distinct spatial scale. With both comprehensive predictive distribution modelling information now at hand, progress can be made on questions about small-size population genetics and on the urgent need for an efficient monitoring programme for the Desman NAP [10]. The endemic vulnerable Pyrenean Desman also appears to be a good example species to address the challenging “rare species modelling paradox” [15] and add to the debates on the biological consequences of increasing “edge effects” (edges becoming proportionately greater relative to core areas following ecosystem fragmentation [37]) and to the contribution of so-called “matrix species” versus “edge-preferring species” to the comprehension of species-area relationships [38].

## Figures and Tables

**Table 1:** Selected variables (in bold) among the initial set of environmental variables with coefficient values of each and order of inclusion in the model (number between bracket).

Variables		Spanish model	French without latitude model	French with latitude model
	Code	(Barbosa et al. [1])	Present study	Present study
**Mean altitude (m)**	**Alti**	**Alti** (6) 0.0021	**Alti** (6) 0.02967	**Alti250** (1) 0.00248
Mean slope (degrees)	Slop			
**Mean annual precipitation (mm)**	**Prec**	**Prec** (1) 0.00077	**Prec** (3) −0.002588	**Prec** (3) −0.003152
Mean relative air humidity in January (%)	HJan			
**Mean relative air humidity in July (%)**	**HJul **	**HJul** (4) −0.10	**HJul** (4) −0.4211	
Mean annual insolation (h/year)	Inso			
**Mean annual solar radiation (kwh/m²/day)**	**SRad **	**SRad** (8) 0.013	**SRad** (2) −153.6	SRad (5) −43.11
Mean temperature in January (°C)	**TJan **	**TJan** (7) 0.43		
Mean temperature in July (°C)	TJul			
**Mean annual temperature (°C)**	**Temp**	**Temp** (2) −0.60	**Temp** (1) 0.3106	
Mean annual number of frost days (min T <0°C)	DFro			
Mean annual potential evapotranspiration (mm)	PET			
Mean annual actual evapotranspiration (mm) (=Min [PET, Prec]	AET			
Maximum precipitation in 24 h (mm)	MP24			
Relative maximum precipitation (= MP24/prec)	RMP			
**Mean annual number of days with precipitation >0.1 mm**	**DPre **	**DPre** (3) 0.0027	**DPre** (5) 0.08299	**DPre** (2) 0.1059
Annual temperature range (°C) (= TJul − TJan)	TRan			
Annual relative air humidity range (°C) (= HJan − HJul)	HRan			
Distance to the nearest highway (km)	DHi			
Distance to the nearest town >100 000 hab (km)	U100			
Distance to the nearest town >500 000 hab (km)	U500			
**Latitude (°N)**	**Lati**	**Lati** (5) 1.13		**Lati** (3) −8.826
Longitude (°E)	Long			
		Constant −44.63	Constant (481)	Constant (496.9)
